# Supplementary material for: Anti-atherosclerotic vaccination against Porphyromonas gingivalis as a potential comparator of statin in mice
Source: PeerJ. 2021 Apr 20;9:e11293. doi: 10.7717/peerj.11293 (PMC8063868; doi:10.7717/peerj.11293)
Supplement: Supplemental Information 2 [file peerj-09-11293-s002.docx]

**ARRIVE 2.0 Checklist**

**Anti-atherosclerotic vaccination against Porphyromonas gingivalis as a potential comparator of statin**

**1. Study design**

a-b) 32 APO E knock out mice were used to carry out two steps of experiment by allocating 8 mice to each of 4 groups at the first step. The four groups with 8 weeks old underwent no treatment (control) and three types of treatments with Pitavastatin, P.gingivalis vaccine, and both for 8 weeks until week 15, followed by one week break (week 16). At the second step, the P.gingivalis challenge was performed by dividing each of four groups with 17 weeks old into two sub-groups (with or without challenge) for the next 5 weeks (Manuscript line 138-148).

**2. Sample size**

a) As described in the study design above, 32 animals were placed four groups (8 mice / group). (Manuscript line 138-148)

b) Sample size analysis

The minimum number of mice to provide statistically significant results will be used. The power analysis is used to calculate the minimum number N at a significance level of 0.01 and power of 0.8. The minimum number N is 4.245 (n=4). (Manuscript line 114-116)

**3. Inclusion and Exclusion criteria**

a) Inclusion criteria and exclusion criteria |

The mice that survived until harvesting aorta without any tissue damage were included. The mice that died or became sick were excluded. Also, the mice in which intact aorta were not extracted were excluded in counting n number.

b) Total 7 mice were excluded due to damage during the processes of aortic extraction, staining, and analysis. (Manuscript line 155-157)

| Excluded mice | Number |
| --- | --- |
| PTV/Pg infection(-) | 1 |
| PTV/Pg infection(+) | 2 |
| vaccine/Pg infection(-) | 1 |
| PTV with vaccine/Pg infection(-) | 1 |
| PTV with vaccine/Pg infection(+) | 2 |

Table 1. Table 1. Excluded mice due to tissue damage

c) The total number of mice included for the final analyses: 25 (=32-7)

**4. Randomization**

a) In order to eliminate any bias from grouping to experiment and to analysis, mouse grouping and experiments were conducted by different people, and mice were randomly assigned to each group (Manuscript line 142).

b) In order to exclude any effects of confounders, the sex and age of the mice were unified, and human errors were minimized via double checking each step of experiments by more than one person.

**5. Blinding**

Mouse grouping and experiments were blinded via conducting by different people, and result analyses were carried out via double checking with more than one person.

**6. Outcome measures**

As described elsewhere, the sample size was determined by power analysis, statistical analyses were carried out to determine significance of each result set, and each result was presented as mean ± standard deviation (Manuscript line 118-157). This study was not driven by a hypothesis but carried out to compare existing drug effects and to validate the effect of combination.

**7. Statistical method**

Analyses were performed using SPSS 23.0 (Systat software, Chicago, IL). One-way ANOVA with Tukey multiple comparisons test was performed to compare the groups. P values less than 0.05 were considered significant. (Manuscript line 160-162)

**8. Experimental animals**

We use Eight-week-old ApoE−/− male mice were purchased from Jackson Laboratories. (Manuscript line 111-112)

**9. Experimental procedure**

We described the details (when, what, where, why, and how) of each experimental procedure in Method (Manuscript line 96-162)

**10. Results**

The sample size was determined by power analysis, statistical analyses were carried out to determine significance of each result set, and each result was presented as mean ± standard deviation for the result figures.

**11. Abstract**

Manuscript line 39-64

**12. Background**

Manuscript line 69-86

**13. Objectives**

Manuscript line 87-92

**14. Ethical statement**

We declare ethical statement in the manuscript (line 108-111).

**15. Housing and husbandry condition**

We have not described all animal testing conditions, and there is a brief description in the manuscript. More additional detailed conditions will be described in the following description. (Manuscript line 111-114)

Irradiated feed was used, and reverse osmosis water purification was allowed to be fed freely. Individual ventilation cage and cage exchange table were used, and all breeding goods were autoclaved or pasteurized. There was a dedicated breeder for each breeding room, and the cage was exchanged once a week, and the number of mice in one cage was set to 4 and raised. Lighting was changed at 8 am and 8 pm, and enrichment (diamond twist) was provided to all cages.

**16. Animal care and monitoring**

a. pain, suffer, distress

For a sufficient period of time, the researcher was familiar with the use of appropriate anesthetics and analgesics, and was skilled in euthanasia. Through this, pain management and euthanasia can be performed in an appropriate and humane way. Also, stress management was attempted through enrichment.

b. There is no unexpected adverse event during study

c. humane endpoint

During the experiment, we evaluated weight change, hair coat change, eye and nose change, posture and movement, and scored in a way that considers euthanasia if the score exceeded a certain score. In addition, anorexia, weight loss, severe organ or systemic symptoms were set as the humane end point.

**17. Interpretation / scientific implication**

a. interpretation – (Manuscript line 224-229)

b. limitation – (Manuscript line 230-235)

**18. Generalisablility/translation**

The significance of this study lies in the fact that vaccination against p.gingivalis demonstrates a promising potential to prevent arteriosclerosis, and further research is expected to be needed. (Manuscript 226-229)

**19. Protocol registration**

We used the atherosclerosis quantification protocol which is described in Lin Y et al. group.
 (Manuscript 153). Any registration was not required for the protocols that were used in this study.

**20. Data access**

The raw data has been uploaded to the supplement data.

**21. Conflict of interest**

The authors declare that they have no competing interests.
